# Robotic Management of Urolithiasis in the Pediatric Population

**DOI:** 10.3389/fped.2019.00351

**Published:** 2019-08-22

**Authors:** Natalia Ballesteros, Zachary A. Snow, Paulo R. M. Moscardi, George A. Ransford, Pablo Gomez, Miguel Castellan

**Affiliations:** ^1^Division of Pediatric Urology, Nicklaus Children's Hospital, Miami, FL, United States; ^2^Dr. Kiran C. Patel College of Osteopathic Medicine, Nova Southeastern University, Fort Lauderdale, FL, United States; ^3^Division of Pediatric Urology, Jackson Memorial Hospital, Miami, FL, United States; ^4^Division of Pediatric Urology, Florida Hospital for Children, Orlando, FL, United States; ^5^Division of Pediatric Urology, Joe DiMaggio Children's Hospital, Hollywood, FL, United States

**Keywords:** robotic, pyelolithotomy, nephrolithotomy, urolithiasis, renal stones, ureteropelvic junction obstruction, pediatric

## Abstract

A variety of surgical techniques exist for the management of urolithiasis. Minimally invasive techniques have replaced open surgery in the last few decades. For complex stone management, robotic-assisted laparoscopic surgery (RALS) has emerged as a safe and feasible alternative in adults. The literature for RALS for urolithiasis (RALS-UL) in the pediatric population is scarce. Herein, we present a review of the literature in both adult and pediatric patients as well as our experience using RALS-UL at our institutions. Special attention is given to the synchronous management of urolithiasis when surgery is performed for other conditions such as ureteropelvic junction obstruction (UPJO), and a supplemental video is provided.

## Introduction

Urolithiasis is a very common condition affecting both men and women of all age groups. When surgical management is indicated, there are a variety of minimally invasive techniques that have gradually replaced open surgery as the preferred approach (1). Although most patients with kidney or ureteric stones are treated via ureteroscopy (URS) and retrograde intrarenal surgery (RIRS), percutaneous nephrolithotomy (PCNL) or extracorporeal shock wave lithotripsy (ESWL); the use of laparoscopic surgery in stone disease has increased over the last two decades (2), and open surgical interventions such as anatrophic nephrolithotomy and ureterolithotomy are now rarely done. Besides the significant laparoscopic skills needed, drawbacks to laparoscopic stone surgery include challenges with ureteral stenting and suturing, limited dissection and intracorporal reconstruction, as well as increased risk of urinary leak (3–6). The use of the robotic platform allows for improved ergonomics and visualization (magnified, three-dimensional) as well as ease of instrument dexterity that most closely imitates the open technique maintaining the advantages associated with minimally invasive surgery. Robotic assisted laparoscopic surgery for urolithiasis (RALS-UL) overcomes most of the issues associated with open or laparoscopic urolithiasis surgery and the training curve, like most skills, is attainable. Robot-assisted pyelolithotomy, robot-assisted ureterolithotomy and robot-assisted flexible URS are now part of the urologist's arsenal for the treatment of large volume stones and become particularly useful in conditions requiring simultaneous reconstruction (1, 7). In the pediatric population the incidence of urolithiasis appears to be increasing globally (8). The standard procedures to treat stone disease in children have been similar as those used in the adult population (9). However, there is very limited data regarding the use of robotic-assisted surgery in the management of pediatric urolithiasis. Lee et al. (10) demonstrated safe and effective use of robotic-assisted pyelolithotomy in 5 adolescent patients with large stone burdens. To our knowledge, this is the only study dedicated to the surgical management of stone disease robotically within the pediatric population. In this literature review, we will highlight the current advances in the field of robotics for stone disease among the general population.

## Materials and Methods

We performed a review of the major studies in adult and pediatric robotic-assisted laparoscopic surgery for urolithiasis (RALS-UL) from 2005 to 2018. Search words included, but were not limited to: robotic, laparoscopic, urolithiasis, stone, pyelolithotomy, nephrolithotomy, and pediatric.

## Results

Since 2005, 22 articles for RALS-UL have been published in the adult population for management of various conditions: concomitant management of UPJO and nephrolithiasis (4), concomitant management of caliceal diverticuli with stones (2), pyelolithotomy (2), pyelolithotomy with nephrolithotomy (1), extended pyelolithotomy (4), ureterolithotomy (1 proximal, 1 distal), anatrophic nephrolithotomy (3), and management of stones when ectopic and other renal anomalies are present (2 pelvic kidney, 1 horseshoe kidney, 1 cross-fused ectopic kidney). Of note, many of these studies did not include detailed information regarding the initial patient stone burden. Additionally, the video section of one journal in 2012 reported a case of anatrophic nephrolithotomy. This video was not available for viewing.

In the pediatric population, an article from 2007 is the only one that describes RALS-UL: 4 pyelolithotomies and 1 concomitant management of UPJO and nephrolithiasis. Furthermore, the video section of one journal in 2014 depicted a technique using intraoperative ultrasound probing during RALS-UL in children. This video and further details of the study were not available for reviewing.

## Discussion

According to the American Urological Association (AUA) and Endourological Society guidelines on adult urolithiasis, when surgical intervention is necessary, URS/RIRS and ESWL are considered to be first-line treatment for kidney stones <2 cm, whereas for > 2 cm, PCNL is the therapy of choice (11). This is also the case for pediatric patients, with the addition that ESWL can be implemented as well when stones are >2 cm (12). Although these guidelines do not include RALS as a standard of treatment for routine patients, they do include open, laparoscopic, or robotic interventions as an option for patients with rare anatomic anomalies and complex stone disease or for those requiring concomitant reconstruction (12). This is further supported by the European Association of Urology (EAU) guideline for the management of urinary stone disease in children which states that open or laparoscopic alternatives may be inevitable in such situations (13). Ample literature has shown that laparoscopic and robotic-assisted approaches to stone disease are both viable options for patients with large stones, abnormal collecting system anatomy, and complex stone burden (14). The earliest report of laparoscopy for the management of stone disease was published in the 1979, when Wickham first documented his results of laparoscopic ureterolithotomy via a retroperitoneal approach (15). Then, over a decade later, clinical trials investigating this minimally-invasive method began to expand largely due to the creation of new laparoscopic technologies and the widespread adoption of laparoscopic surgical skills (2). In 1994, Gaur et al. (16) demonstrated the safe and effective use of retroperitoneal laparoscopic pyelolithotomy in 5 patients with medium-sized pelvic stones not amenable to ESWL or PCNL. Since then, multiple studies have been performed showing that laparoscopic pyelolithotomy is comparable to PCNL for kidney stones > 2 cm in the renal pelvis, with certain advantages such as less blood loss and less post-operative analgesia requirement (14, 17). As for laparoscopic ureterolithotomy, clinical trials comparing this approach to traditional flexible ureteroscopy for large upper tract stones (> 2 cm) show comparable to higher stone-free rates, less need for any additional procedures and lower complication rates (18). The success of conventional laparoscopic techniques in the management of large volume stones in combination with the development of the da Vinci® Surgical System by Intuitive Surgical Inc. eventually gave way to the use of robotic-assisted surgery in the treatment of urolithiasis. The use of RALS-UL was first reported by Atug et al. (19). Since then, RALS-UL has become a great tool in the urologist armamentarium for managing diverse complex and challenging cases of urolithiasis where other known modalities may not be feasible or when multiple prior procedures have been attempted.

### General Considerations

#### Preoperative Urine Culture and Antibiotics

The importance of documented urine sterilization prior to urologic surgical intervention cannot be overstated, especially in the setting of intraperitoneal and/or retroperitoneal urine spillage during robotic cases (20). Likewise, perioperative antibiotic prophylaxis should be used based on urologic guidelines and preoperative culture sensitivity.

#### Ureteral Stents

In general, placement of ureteral double J (JJ) stents is performed depending on the case and at the surgeon's discretion. They can be conducted antegrade during the procedure in similar fashion as when placed in open cases. In other instances, stents may have been already placed preoperatively to relieve obstruction. For other cases, such as in anatrophic nephrolithotomies, routine ureteral stent placement is not done (20). At one of our institutions, we prefer to perform a retrograde pyelogram (RPG) and JJ stent placement prior to all RAL pyeloplasties; therefore, it is done in this manner for concurrent management of renal stones. At another institution (FHC), retrograde pyelogram is not routinely performed and urinary drainage (JJ stenting or nephrostomy tube) is avoided in the majority of RAL pyeloplasties. Indications for JJ stenting at this institution include massive reduction of the renal pelvis, solitary kidney and age <6 months. Additionally, for isolated pyelolithotomies, we do not leave a ureteral stent when the ureteropelvic junction (UPJ) is not compromised. On infants, we place 3.7 Fr stents, and in older children we use either 3.7 or 4.8 Fr stents.

#### Nephroscopy and Stone Retrieval

**Figure 1:**
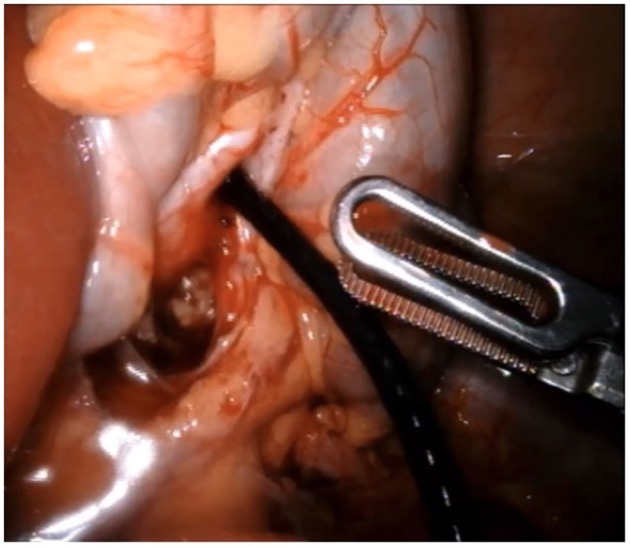
Nephroscopy using a standard digital flexible ureteroscope.

Regardless of stone location in the upper collecting system, the use of the flexible or rigid ureteroscope is a great addition to the surgical intervention. The scope is deployed through an assistant port, when available, or through a robotic trocar after one of the arms is undocked. A ureteral access sheath can be telescoped through the trocar if multiple stones are found or if a large stone burden warrants it. This maneuver decreases surgical time. At our institutions we prefer the use of a flexible ureteroscope for nephroscopy when multiple stones are present to maximize stone clearance (Figure 1). The stones can be retrieved with the ProGrasp forceps (Intuitive Surgical INC, Sunnyvale, CA, USA), when easily visible, or using endoscopic graspers or baskets. Laser lithotripsy can be employed as necessary to break large stones into smaller fragments that are easier to retrieve (21). Furthermore, for removing stones from the abdominal cavity, simultaneous removal through the trocar or the use of a commercially available or homemade bag is possible. One article mentioned the use of the fingers of a sterile glove as retrieval bags (21). Interestingly, the use of a flexible cystoscope has also been described to ease intraperitoneal stone removal after pyelolithotomy is closed (22).

#### Intraoperative Ultrasonography

The use of the laparoscopic ultrasound probe is an additional tool that can aid in localizing calculi before or after nephroscopy and can also be used to identify hilar vessels when needed, for example in anatrophic nephrolithotomy cases (20, 23, 24).

#### Type of Suture

The collecting system is closed with interrupted or continuous absorbable suture depending on surgeon's preference. In cases of nephrolithotomy, the renal parenchyma can be closed with braided or barbed absorbable suture.

#### Drain Placement

In many cases, a JP or similar suction drain has been left intraperitoneally for 1–2 days (24–26). At our institutions, for robotic pyeloplasties and other pediatric surgeries, we do not traditionally leave such drain and instead we leave a Foley catheter for 1–2 days. If a JJ stent is left, it is removed 4–6 weeks later. For cases of RALS-UL, we have not had urinary leaks or other complications in this manner.

### Urolithiasis and Concomitant UPJO

When presented with cases of UPJO and renal stone disease, options for surgical management are limited. The European Association of Urology guidelines currently recommend that robotic-assisted pyeloplasty (RALP) and surgical intervention for stones be performed in separate procedures (27). However, due to the potential risks of general anesthetics on the brain development of children undergoing multiple and lengthy procedures (28, 29), one should consider as an alternative to perform both surgeries concurrently. Laparoscopic pyeloplasty with pyelolithotomy with or without flexible URS to completely clear stone burden has been widely described in the adult literature. Concomitant RALP and pyelolithotomy (RALP +P) in these patients was first reported by Atug et al. (19). After pyelotomy, flexible renoscopy with stone removal was achieved on 8 patients with 100% stone clearance confirmed on imaging. Since then, 3 more studies looking specifically at RALP + P have emerged supporting the safety and feasibility of the procedure along with high stone-free rates (27, 30, 31). Of these, Zheng et al. (27) reported their experience removing renal calculi with a rigid ureteroscope in 9 patients. The stone-fee rate was 89% with one patient requiring one ESWL session for complete stone clearance. Most recently, in 2017 Jensen et al. (30) demonstrated that although the median operative time for concomitant RALP and RALS-UL was 31 min more than RALP alone, no statistical difference in the blood loss or length of stay were observed.

**Figure 2:**
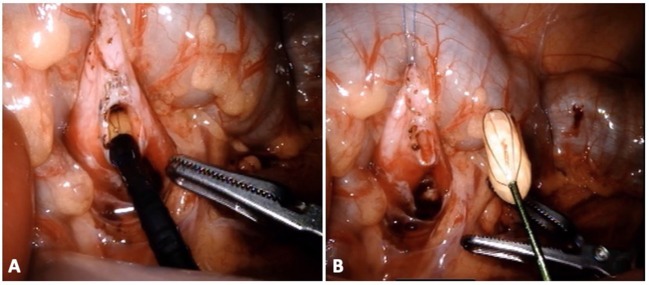
Patient with UPJO and nephrolithiasis undergoing pyeloplasty and concomitant nephrolithotomy. Through the flexible ureteroscope **(A)**, a standard basket device is used for stone extraction **(B)**.

In the pediatric population, only one case has been described of concomitant RALP and pyelolithotomy on an adolescent. Further details regarding the stone burden was not reported but the patient was rendered stone free with a single procedure (10). At our institutions, over the last 5 years we have performed concomitant RALP + P on 10 patients from 5 to 26 years of age (supplementary video: https://doi.org/10.6084/m9.figshare.8799269.v1). All cases had preoperatively been diagnosed with UPJO and ipsilateral nephrolithiasis. As above mentioned, after pyelotomy, nephroscopy and stone removal was performed (Figure 2). Length of stay was 1 day in 7 cases. The extra day spent by the remaining patients was for pain control or for observation. Nine of 10 patients were rendered stone free with robotic intervention. In 2 patients, no stone was found. One of them had a punctate stone recognized on preoperative computed tomography (CT) and was believed to have been flushed out with irrigation as it was not visualized later on postoperative imaging. The other patient had a history of a small lower pole calculus. This was missed during nephroscopy and shortly after the pyeloplasty he passed the stone spontaneously. Of note, this patient later underwent other interventions for recurrent urolithiasis, including on the contralateral kidney.

### Pyelolithotomy, Extended Pyelolithotomy, and Nephrolithotomy

**Figure 3:**
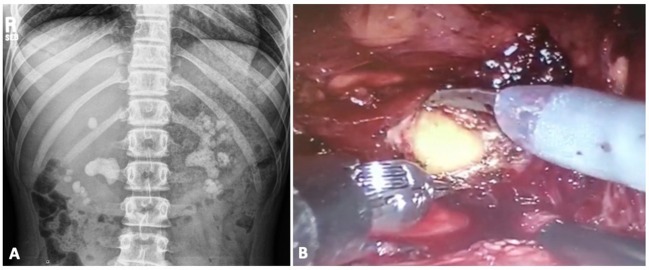
Patient with bilateral staghorn calculi and cystinuria **(A)** undergoes metachronous bilateral pyelolithotomies **(B)**.

With the above-mentioned success of concurrent RALP + P, robotic pyelolithotomy has become the most common approach in the RALS-UL literature and further applications have been explored to include nephrolithotomy and extended pyelolithotomy procedures. Pyelolithotomy involves incision of the renal pelvis with subsequent stone extraction. Further involvement of the calyceal system calls for an extended approach. Nephrotomy and nephrolithotomy is performed to allow stone extraction when there is a narrow infundibulum or part of the calculus is attached to the parenchyma. In 2006, Badani et al. (32) reported their experience with robotic extended pyelolithotomy (REP). The patient is positioned in modified lateral decubitus with minimal to no flexion and without kidney rest elevation. After adequate exposure of the renal pelvis, a pyelotomy is performed away from the UPJ. The authors demonstrated complete stone removal and minimal blood loss in 12 patients with partial staghorn calculi. Of note, a 13th patient with complete staghorn stones was unsuccessfully cleared, leading to the conclusion that REP is not a suitable technique for full staghorn calculi. Other authors have described REP with similar experiences (33–35), with the latest one being a case report of a 31-year-old non-diabetic woman with emphysematous pyelonephritis caused by on a 6.5 cm gas-containing calculus (35). In 2015, Rajiv et al. (25) described a case of bilateral large stone disease managed with simultaneous bilateral robot-assisted pyelolithotomy, showing minimal morbidity, a short hospital course and the avoidance of adjuvant procedures. A multi-center evaluation of robotic pyelolithotomy and robotic nephrolithotomy in 27 patients between 2008 and 2014 concluded that both techniques are safe (minimal bleeding and low risk of sepsis) and effective (maximum stone free clearance and infrequent retreatment rates) for patients with large renal pelvis and calyceal stones (36). Cystine stones account for 1–2% and 6–8% of urolithiasis in the adult and pediatric population, respectively (21). Although cystinuria is best treated medically for stone prevention, the surgical management of large stones can be quite a challenge and may require multiple endourologic procedures. On the other hand, robotic pyelolithotomy is a feasible alternative. In 2017, Megiatto et al. (21) reported a case of a 20-year-old woman who was poorly compliant with medical treatment and had undergone several procedures through the years for her disease. On one side, there was a single stone that required both URS and ESWL to clear. For her large, multiple stone burden contralaterally (36 stones), she underwent robotic pyelolithotomy with concomitant renoscopy and laser lithotripsy. In the only published report of RALS-UL in the pediatric population, 4 patients, between the ages of 10 and 23, underwent robotic-assisted laparoscopic lithotomy for the treatment of large cystine stones 2–7 cm in size (10). These stones had been refractory to previous PCNL and ESWL. Additionally, one patient required open conversion due to an intrarenal pelvis and inability to remove the stone. This patient required 3 additional procedures (ESWL and 2 URS) to become stone free. In 2014, Ghani et al. (23) published a video that depicts a technique for using intraoperative ultrasound probing during RALS-UL in 4 children to aid in stone localization. This video was not available for viewing but is worth mentioning as an additional tool that can be applied to RALS-UL. At one of our institutions, we performed metachronous bilateral pyelolithotomies in a 15-year-old girl with bilateral staghorn calculi and a new diagnosis of cystinuria (Figure 3). In both cases, no ureteral stents were left indwelling as the UPJs were not compromised. She had a 1-night hospitalization per each surgery. There were no overall complications and she was 100% stone free following the procedures.

### Calyceal Diverticular Calculi

Another example of approaching urolithiasis concomitantly with the management of another condition is in the treatment of calyceal diverticular calculi. The incidence of stones within a calyceal diverticulum ranges from 1 to 10% (37). Although these are managed primarily with ESWL, RIRS and PCNL; laparoscopic and robotic assisted interventions are feasible alternatives. Furthermore, laparoscopic intervention has been proposed to be the approach for management of anteriorly-located calyceal diverticuli that have failed prior endourologic attempts or for anteriorly-located stones > 3 cm (38). In 2014, Torricelli et al. (37) reported the first case managed robotically in a 33-year old obese woman with a symptomatic 2 cm, anterior mid polar calyceal diverticular calculus. She had undergone two prior failed URS attempts: one with diverticular laser incision but inability to remove stone fragments after lithotripsy and the second had found the diverticular neck to be obliterated. After RALS-UL, the diverticulum was managed with fulguration. The patient was in a 45-degree lateral decubitus position with the table flexed, and the ports were placed in similar fashion as for robotic-assisted laparoscopic partial nephrectomy (RALPN). As in RALPN, the renal vasculature was clamped for warm ischemia. Likewise, an intraperitoneal drain was left in place for 2 days. More recently, Verbrugghe et al. (39) described their similar experience with an anterior calyceal diverticulum with stone burden in the lower pole of a 55-year old woman with an additional history of pyelonephritis. In the first case, the use of a robotic ultrasound probe was used for identification of the diverticulum while in the second case a CT-guided puncture of the calyx was done with a harpoon left in place for localization.

### Congenital Renal Anomalies

Ectopic pelvic and horseshoe kidneys are a surgical challenge in the management of ureterolithiasis. They are located in close proximity to the bony pelvis and are surrounded by important visceral organs. In addition to abnormal vasculature, they have intrinsic anatomical anomalies such as malrotation, anteriorly displaced renal pelves, and high ureteral insertions. These often lead to partial or complete urinary obstruction and hydronephrosis. UPJO is found in 70% of ectopic kidneys with hydronephrosis (5). Concomitant urolithiasis poses a challenge for endourological interventions, including PCNL requiring modified renal access. RALS-UL is an alternative that has been demonstrated in the literature to be safely feasible. Pyeloplasty with concomitant pyelolithotomy on an ectopic pelvic kidney with UPJO and nephrolithiasis was first described in 2010 by Zheng et al. (27). The patient was a 55-year old man who presented with abdominal pain, a serum creatinine (Cr) of 2.3 mg/dL, and the aforementioned renal anomalies. Six months after surgical intervention, the patient was doing well, Cr had significantly improved, and renal function had increased from 19 to 24%. In the management of isolated large-burden urolithiasis in these anomalous kidneys, the traditional surgical modalities have been employed in the past such as RIRS, ESWL, and PCNL. However, due to the above-mentioned anatomic challenges, these surgeries are often difficult and unsuccessful for complete stone clearance (24). In these cases, endopyelotomy with subsequent pyelolithotomy can be accomplished. Examples described in the literature include an ectopic pancake, or fused lumped kidney, with a 2.5 cm stone in one of the moieties (40) and a horseshoe kidney with multiple pelvicalyceal calculi (25). In most cases, patient positioning and robotic port placement were done in a prostatectomy-like configuration. In the pediatric population, this would be comparable to positioning and port placement for bilateral vesicoureteral reimplantations. A transmesenteric approach was utilized for endopyelotomy and subsequent pyelolithotomy thus avoiding mobilization of the colon and its increased operative time and potential risk for injury (22, 25).

### Ureterolithiasis

RALS-UL is also helpful in the setting of complex ureteral calculi and prior surgical interventions. It's use in the upper urinary tract with good outcomes has been previously reported (41). In 2015 Olvera-Posada et al. (3) reported the case of a 66-year-old female with a history of recurrent ureterolithiasis, recurrent urinary tract infections (UTIs), two large obstructing right proximal ureteral calculi, and a right split renal function of 37%. Despite a remote successful ureteroscopy in the past on that same kidney, the patient underwent failed attempts at antegrade or retrograde access to the stone in this instance; partially due to ureteral tortuosity and significant inflammatory changes. She underwent robotic assisted ureteropyelostomy for the stone removal followed by pyeloplasty for closure. At 1-year follow up renogram the right renal function was 42% and there was good drainage. Likewise, RALS-UL has been performed successfully in the lower ureters. In 2013 Dogra et al. (4) published outcomes on 16 patients (mean age 27) treated with robotic-assisted distal ureterolithotomies for impacted stones over 2 cm in size. Their mean operative time was 45.3 min with a mean console time of 20.3 min, which was significantly shorter when compared with a traditional endoscopic approach. Additionally, they demonstrated greater clearance rates and a lower incidence of complications compared to URS. Of note, all patients underwent urethral catheterization, intraperitoneal ureteral stenting, and placement of intraperitoneal drain. The drain was removed on postoperative day (POD) 1 and the Foley on POD 2, with the ureteral stents removed around 4 weeks later.

### Robotic Anthropic Nephrolithotomy

Open anatrophic nephrolithotomy is done now rarely despite its high rate of stone clearance due to the morbidity of the procedure. In the majority, the laparoscopic method has been performed with warm ischemia, due to the difficulties achieving renal icing intraperitoneally (24). This modality is not ideal for preservation of renal function. Sotelo (42) was the first to report a case of robotic-assisted anatrophic nephrolithotomy (RANL). This was done via video submission and it was not available for viewing at this time. They reported implementation of vascular control with early unclamping and controlled hypotension. Like in the laparoscopic approach, warm ischemia was replicated with a time of 26 min. The patient required intraoperative blood transfusion, a residual 1 cm stone was later identified, and furosemide renogram showed 10% renal function loss at 1 month of follow up. In 2013, using their practice with cold ischemia for partial nephrectomies, Ghani et al. (24) then reported their experience performing RANL on three patients. They described their technique which includes initial cystoscopic placement of a JJ ureteral stent and the patient is then placed in a traditional flank position. Port placement includes a gel port placed in the midline, with a camera and assistant port through it, two more robotic arm ports and an additional assistant port. Renal hypothermia was achieved with ice slush which was introduced through the gel port. Following this, Mannitol was administered, and the hilum was clamped. The incision was made through the avascular plane and after nephrolithotomy, the collecting system was closed with monofilament suture and running barbed suture was used for the renal parenchyma. The mean cold ischemia time was 57 min. The authors achieved a stone-free rate of 33%, with intraoperative knowledge that 2 of the 3 patients had incomplete stone clearance, despite having used intraoperative ultrasonography. These 2 patients required PCNL to remove the residual calculi. One patient had a drain placed as a nephrostomy tube for drainage of pyonephrosis at the time of stone removal. There were no complications including no need for blood transfusion. Renal function preservation was corroborated upon follow up based on creatinine clearance. Another study of RANL in 2014 was performed by King et al. (26) on 7 patients, 5 with complete staghorn calculi. The mean warm ischemia time was 35 min. They demonstrated a 29% stone-free rate with minimal hospital length of stay and few post-operative complications, including a blood transfusion and continuous bladder irrigation in one patient for hematuria. Although these studies failed to demonstrate significant stone-free rates as compared to the open technique, both studies highlight the successful debulking of large staghorn stones in a traditionally difficult to manage population and with improved morbidity. Also, further research needs to be done to determine if there is a significant long-term difference in renal function with regards to the use of cold vs. warm ischemia. Another advantage of performing this surgery robotically, is the application of intraoperative near infrared fluorescence (NIRF) with indocyanine green (ICG) dye. Although not performed in humans, NIRF has been assessed in porcine models to detect the avascular renal plane for proper demarcation of the site of nephrolithotomy (43). Madi et al. (20) described their use of NIRF intraoperative to assess if there is any residual renal perfusion after vascular clamping perhaps from an accessory or unclamped vessel. They do not mention if a dose of ICG was given before obtaining vascular control for use on the kidney to identify the avascular plane.

## Conclusion

Minimally invasive management of stones in general has evolved in the last few decades.

Although URS, RIRS, ESWL, and PCNL are still first line treatment in the management of pediatric and adult urolithiasis, RALS-UL has been demonstrated to be safe, effective, and with high stone-free rates in some specific cases. Although all authors stated encouraging results, no study compared the robotic approach to first-line minimally-invasive surgery. RALS-UL is a viable option for patients in whom the above minimally invasive techniques are not applicable or in circumstances where their use is hampered: complex urinary tract calculi, failed prior procedures, or abnormal genitourinary anatomy. Furthermore, concomitant RALS-UL can be performed when other reconstructive interventions are planned. For example, RALP + P should constitute the first choice of treatment for concomitant renal stones and UPJO. The use of flexible URS aids in localizing and extracting stone burden. We have successfully performed RALS-UL at our institution and though the pediatric literature is scarce, this area continues to be one that needs further research.

## Author Contributions

NB and ZS contributed to the conception and design of the study. NB, GR, and PG contributed to database acquisition and analysis. NB and ZS co-wrote the first drafts of the manuscript. PM, PG, and MC wrote sections of the manuscript. All authors contributed to manuscript revision, read and approved the submitted version, and agree to be accountable for all aspects of the work.

### Conflict of Interest Statement

The authors declare that the research was conducted in the absence of any commercial or financial relationships that could be construed as a potential conflict of interest. The reviewer MG is currently organizing a research topic with one of the authors MC and confirms the absence of any other collaboration.
